# Deficiency of Angulin-2/ILDR1, a Tricellular Tight Junction-Associated Membrane Protein, Causes Deafness with Cochlear Hair Cell Degeneration in Mice

**DOI:** 10.1371/journal.pone.0120674

**Published:** 2015-03-30

**Authors:** Tomohito Higashi, Tatsuya Katsuno, Shin-ichiro Kitajiri, Mikio Furuse

**Affiliations:** 1 Division of Cell Biology, Department of Physiology and Cell Biology, Kobe University Graduate School of Medicine, Kobe, Japan; 2 Department of Otolaryngology—Head and Neck Surgery, Graduate School of Medicine, Kyoto University, Kyoto, Japan; 3 Division of Cerebral Structure, National Institute for Physiological Sciences, Okazaki, Aichi, Japan; 4 Department of Physiological Sciences, SOKENDAI (The Graduate University for Advanced Studies), Okazaki, Aichi, Japan; Emory University School of Medicine, UNITED STATES

## Abstract

Tricellular tight junctions seal the extracellular spaces of tricellular contacts, where the vertices of three epithelial cells meet, and are required for the establishment of a strong barrier function of the epithelial cellular sheet. Angulins and tricellulin are known as specific protein components of tricellular tight junctions, where angulins recruit tricellulin. Mutations in the genes encoding angulin-2/ILDR1 and tricellulin have been reported to cause human hereditary deafness DFNB42 and DFNB49, respectively. To investigate the pathogenesis of DFNB42, we analyzed mice with a targeted disruption of *Ildr1*, which encodes angulin-2/ILDR1. *Ildr1* null mice exhibited profound deafness. Hair cells in the cochlea of *Ildr1* null mice develop normally, but begin to degenerate by two weeks after birth. Tricellulin localization at tricellular contacts of the organ of Corti in the cochlea was retained in *Ildr1* null mice, but its distribution along the depth of tricellular contacts was affected. Interestingly, compensatory tricellular contact localization of angulin-1/LSR was observed in the organ of Corti in *Ildr1* null mice although it was hardly detected in the organ of Corti in wild-type mice. The onset of hair cell degeneration in *Ildr1* null mice was earlier than that in the reported *Tric* mutant mice, which mimic one of the tricellulin mutations in DFNB49 deafness. These results indicate that the angulin-2/ILDR1 deficiency causes the postnatal degenerative loss of hair cells in the cochlea, leading to human deafness DFNB42. Our data also suggest that angulin family proteins have distinct functions in addition to their common roles of tricellulin recruitment and that the function of angulin-2/ILDR1 for hearing cannot be substituted by angulin-1/LSR.

## Introduction

Tight junctions (TJs) contribute to epithelial barrier function by eliminating extracellular spaces between adjacent epithelial cells to restrict the leakage of solutes and fluids through the paracellular pathway [1]. By freeze-fracture electron microscopy, TJs are visualized as a set of fibril-like structures, known as TJ strands, circumscribing the cell as a belt [2]. Claudin family membrane proteins are the main component of TJ strands and are directly involved in the function of TJs [1,3,4,5]. To date, mutations of several claudin genes have been identified as causes of hereditary diseases and knockout mouse models of many claudin genes have been reported to exhibit disease or lethality [5,6], demonstrating that the regulation of paracellular permeability is crucial for normal functions of various organs.

At tricellular contacts (TCs), where the vertices of three polygonal epithelial cells meet, TJs form specialized structures, known as tricellular TJs (tTJs): the uppermost horizontal TJ strands formed between each pair of three cells turn to and extend in the basal direction at TCs [2]. Consequently, long and narrow tubes are formed at the extracellular space of TCs and these structures are thought to impede free diffusion of solutes [2]. To date, two types of integral membrane proteins, tricellulin [7] and angulin family proteins [8,9], are known to be molecular components of tTJs. Tricellulin belongs to tight junction-associated MARVEL protein (TAMP) family [10] and has four transmembrane domains. Tricellulin is expressed ubiquitously in various types of epithelial cells [7]. Angulin family proteins, including lipolysis-stimulated lipoprotein receptor (LSR), immunoglobulin-like domain containing receptor (ILDR)1 and ILDR2, are type-I transmembrane proteins with an extracellular immunoglobulin-like domain [9]. Because of their common structures and functions as tTJs-associated membrane proteins, we previously proposed to designate LSR, ILDR1 and ILDR2 as angulin-1, angulin-2 and angulin-3, respectively [9]. Thus, we use a nomenclature of angulin-1/LSR, angulin-2/ILDR1 and angulin-3/ILDR2 for angulin family proteins in this study. The angulin subtypes are expressed complementarily in many epithelial cell types although angulin-1/LSR and angulin-2/ILDR1 are co-expressed in some regions [9]. Previous studies using cultured epithelial cells showed that tricellulin and angulins are required for full barrier function of epithelial cells with high transepithelial electrical resistance [7,8,9,11]. Importantly, angulins recruit tricellulin to TCs through direct or indirect interaction between the cytoplasmic domain of angulins and the C-terminal cytoplasmic domain of tricellulin [8,9].

Recently, it has been recognized that tTJs are necessary for normal hearing [12,13]. Recessive mutations in *TRIC*, which encodes tricellulin, cause a non-syndromic hereditary deafness DFNB49 (MIM 610153) [14]. Consistently, knock-in mice harboring a nonsense mutation of *Tric* encoding a truncated tricellulin (*Tric* ^*R497X/R497X*^ mice), which mimics one of the mutations observed in the DFNB49 pedigrees, exhibited congenital profound deafness associated with degeneration of hair cell in the cochlea of the inner ear [15]. Moreover, recent reports showed that mutations of *ILDR1*, which encodes angulin-2/ILDR1, cause another non-syndromic hereditary deafness DFNB42 (MIM 609646) [16]. Immunofluorescence staining revealed that angulin-2/ILDR1 is expressed in the cochlea together with angulin-1/LSR. In the organ of Corti, angulin-2/ILDR1 is abundantly expressed, while TC localization of angulin-1/LSR is hardly detected. Angulin-1/LSR is detected at TCs in stria vascularis, and angulin-3/ILDR2 is undetectable in the inner ear [9]. Using a cultured epithelial cell line in which angulin-1/LSR expression is knocked-down and no endogenous angulin-2/ILDR1 or angulin-3/ILDR2 is expressed, we have shown previously that most of the mutations associated with DFNB42 abolish or weaken the localization of angulin-2/ILDR1 and recruitment of tricellulin at TCs [9].

In this study, we analyzed an *Ildr1* null mouse lacking angulin-2/ILDR1 in terms of hearing ability, histology of the inner ear and the relationship between angulin-2/ILDR1 and tricellulin. We report that *Ildr1* null mice exhibit deafness and postnatal hair cell degeneration in the cochlea. We also show that tricellulin localization remains at TCs in the organ of Corti in *Ildr1* null mice, with compensated up-regulation of angulin-1/LSR. These findings indicate that angulin-2/ILDR1 has a specific function, which cannot be substituted by angulin-1/LSR in hearing.

## Materials and Methods

### Animals and genotyping

**Fig 1:**
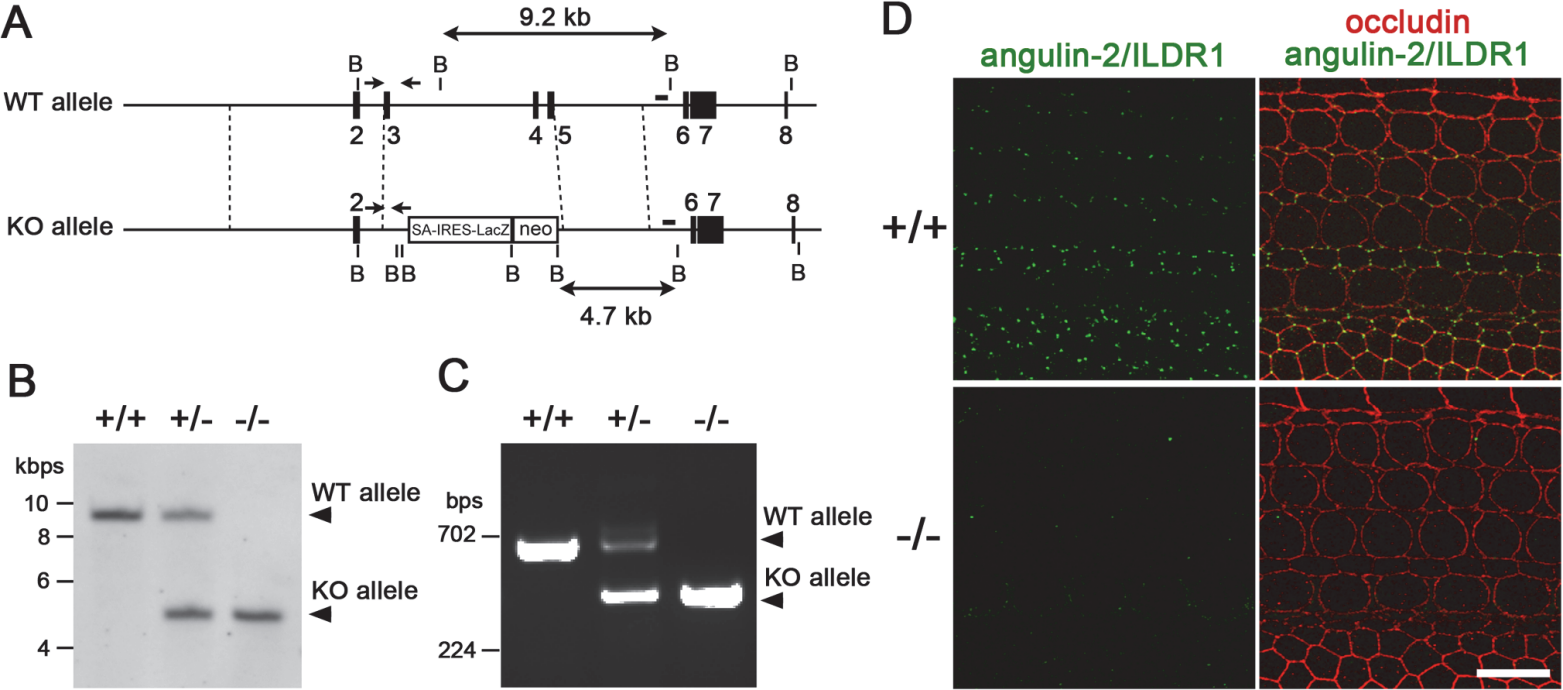
Confirmation of ILDR1/angulin-2 gene targeting in *Ildr1* ^*k-/-*^ mice. A. Restriction maps for wild-type (WT) and targeted (KO) allele. Exons 3, 4 and 5 are replaced with a splicing acceptor-IRES-LacZ (SA-IRES-LacZ) and neomycin-resistance gene (neo) cassette in the KO allele. The position of the probe for Southern blotting and primer pairs for genotyping PCR are indicated as a bar and arrows, respectively. B, *BamH I*. B. Genotype analyses by Southern blotting of *BamH I*-digested genomic DNA from wild-type (+/+), heterozygous (+/-) and homozygous (-/-) mice for the mutant ILDR1 gene allele. Southern blotting with the probe indicated in A yielded a 9.2- and 4.7-kbp band from WT and KO allele, respectively. C. Genotype analyses by PCR amplification of genomic DNA. Primer pairs indicated in (A) yielded a 680- and 388-bp band from WT and KO allele, respectively. D. Loss of the angulin-2/ILDR1 protein in the organ of Corti in *Ildr1* ^*k-/-*^ mice at P3 examined by immunofluorescence microscopy. The tissue was double-stained with anti-angulin-2/ILDR1 pAb (green) and anti-occludin mAb (red). In the wild-type tissue, angulin-2/ILDR1 was concentrated at TCs of hair cells and supporting cells, whereas in the *Ildr1* ^*k-/-*^ tissue these signals became undetectable. Bar, 10 μm.

Mice carrying a deletion in *Ildr1* exons 3, 4 and 5 (*Ildr1* ^*tm1(KOMP)Wtsi*^) (Fig. 1A) were obtained from the Wellcome Trust Sanger Institute Mouse Genetics Project (Sanger MGP). The mouse strain is generated in Sanger MGP using the ES cell clone (EPD0384_1_C10) from the Knockout Mouse Project (KOMP) (Project ID CSD31366). The proper recombination of the genome was confirmed by Southern blotting of *BamH I*-digested genome using a probe located outside of 3’-homologous arm. The DIG-labeled probe was amplified by a specific primer pair (5’-AGTGCTGTGGGTTGGTATTAAG-3’ and 5’-TCTATTCTTCTCCTTAACTAGC-3’) to generate a 600 bp fragment and detected by alkaline phosphatase-labeled anti-DIG antibody and CSPD (Roche, Basel, Switzerland). Genotyping was performed using PCR with a specific primer pairs (common, 5’-CGAAGGAATCTTTCCAAATTGAGGC-3’; WT, 5’-ATCCATAGACCAAGTTCCAGGGAAG-3’; KO, 5’-AGGAACTTCGGAATAGGAACTTCGG-3’) at 5’-arm.

### Animal experiments

Experiments with mice for histological studies were approved by the Kobe University Animal Care and Use Committee (Permit number: P130909) and conducted in accordance with the Regulations for Animal Experimentation of Kobe University. Experiments with mice for behavioral studies were approved by the Animal Research Committee of Kyoto University Graduate School of Medicine (Permit number: 11179) and conducted in accordance with the National Institutes of Health Guidelines for the Care and Use of Laboratory Animals. Anesthesia of mice was performed with midazolam and xylazine. Euthanasia of mice was performed by cervical dislocation.

### Antibodies

The rat anti-occludin monoclonal antibody (mAb), rabbit anti-occludin polyclonal antibody (pAb) [17], rat anti-angulin-1/LSR mAb, rabbit anti-angulin-2/ILDR1 pAb [9], and rat anti-tricellulin mAb (24–69) [7] were characterized as described previously. The rabbit anti-cleaved-Caspase-3 (Asp175) pAb (# 9661) was purchased from Cell Signaling Technology (Danvers, MA, USA). Alexa Fluor 546-phalloidin (#A22283), Alexa Fluor 488 goat anti-rabbit IgG pAb (#A11008) and Alexa Fluor 546 goat anti-rat IgG pAb (#A11081) were purchased from Life Technologies (Waltham, MA, USA).

### Auditory-brainstem-response (ABR) measurements

ABR measurements were performed using P35 mice (n = 4 *Ildr1* ^*k-/-*^, n = 3 wild-type) in a soundproof room as described previously [18]. Tone Bursts of 10, 20 and 40kHz pure tones (rise time 0.1ms, flat time 0.8ms, fall time 0.1ms) were used to evoke ABR (system 3; Tucker-Davis Technologies, Alachua, FL). The ABR waveforms were recorded for 12.8 ms at a sampling rate of 40,000 Hz using 50–5,000-Hz filter settings; waveforms recorded from 1,024 stimuli at a frequency of 9 Hz were averaged. ABR waveforms were recorded in decreasing 5-dB SPL intervals from the maximum amplitude until no waveforms could be visualized. Statistical analysis of the data was performed using Student’s t test of independent variables (2-tailed distribution) as previously described [15].

### Immunofluorescence microscopy

For immunofluorescence microscopy of mouse inner ear tissues, the inner ears from mice at P3, P10, P12, P15 and P35 were dissected and fixed for 20 min by injection of 10% (w/v) trichloroacetic acid (TCA) in water or 4% (w/v) formaldehyde in PBS through oval and round windows. After 20 min, the tissues were washed with PBS, decalcificated with 10% (w/v) EDTA for 3 days in PBS except for P3 mice, microdissected into the organ of Corti and permeabilized by incubation with PBS containing 0.2% (w/v) Triton X-100 for 30 min at room temperature. The samples were blocked with 2% bovine serum albumin (BSA) in PBS and incubated with primary antibodies followed by the labeling with fluorescence-labeled secondary antibodies. The samples were embedded in Fluoromount-G (SouthernBiotech, Birmingham, AL, USA) and observed with laser confocal microscope (model SP8; Leica microsystems, Wetzlar, Germany).

The compensatory localization of angulin-1/LSR in *Ildr1* ^*k-/-*^ mice was analyzed with ImageJ software. Using stacked confocal images of ~10 μm in depth, the fluorescence intensity of angulin-1/LSR or occludin within a circle of 0.45-μm radius from the center of TC delineated by occludin staining was measured. The fluorescence intensity within a circle of 0.45-μm radius in the cytoplasmic region at 0.5–1 μm apart from each TC was set as the background, and subtracted from each raw fluorescence intensity. The mean fluorescence intensity from 100 TCs in typical images from two wild-type mice or two *Ildr1* ^*k-/-*^ mice was calculated for angulin-1/LSR and occludin. The compensatory localization of angulin-1/LSR or occludin at TCs was determined by comparing the average fluorescence intensity in *Ildr1* ^*k-/-*^ mice with that in wild-type mice.

### Scanning electron microscopy

The samples from mice at P3, P10, P12, P15 and P35 were obtained in the same way as immunofluorescence microscopy except for the pre-fixation with PBS containing 2% glutaraldehyde for 2 hr instead of TCA or formaldehyde fixation. After microdissection to organ of Corti, the tissue was dehydrated with a dilution series of ethanol (50%, 60%, 70%, 80%, 90%, 99% and 100%), immersed in t-butanol, frozen at -20°C and then the t-butanol was sublimated off. The sample was then sputter-coated with platinum-palladium alloy using an ion coater (model IB-3; Eiko, Tokyo, Japan) and observed with a scanning electron microscope (model S-4700; Hitachi, Tokyo, Japan).

### Toluidine blue-staining of semi-thin sections of the cochlea

The samples were obtained in the same way as scanning electron microscopy. The fixed cochleae were dehydrated with a dilution series of ethanol (65%, 75%, 85%, 95%, 99% and 100%) and propylene oxide and were embedded in Epon 812 resin. Semi-thin sections of 3-μm thickness were cut with a diamond knife and then stained with toluidine blue. Samples were observed with an upright microscope (model BX41, Olympus, Tokyo, Japan) equipped with a digital camera.

### FM1-43 uptake

Cochleae were dissected from P3 *Ildr1* ^*k-/-*^ and wild-type mice in PBS and explanted onto a glass-bottom dish (MatTek, Ashland, MA, USA) previously coated with 20-fold diluted matrigel (BD Biosciences). Explants were incubated in DMEM supplemented with penicillin G at 37°C with 5% CO_2_ for 12 hours. FM1-43 (Life Technologies) was applied to the mounted tissue according to the method previously described with some modifications [19]. Explants were washed 3 times with HBSS (nacalai tesque) containing 10 mM HEPES buffer and 1.3 mM Ca^2+^ (HEPES-HBSS-Ca). After an additional 20 minutes incubation in HEPES-HBSS-Ca, 5 μM FM1-43 in HEPES-HBSS was applied for 10 seconds at room temperature (23~25°C), followed immediately by 4 time washes (within 1 minute) with HEPES-HBSS-Ca. When testing the effects of BAPTA, HEPES-HBSS-Ca was replaced with HEPES-HBSS containing 5 mM BAPTA in every step. FM1-43 fluorescence from magnified images from middle turns of cochleae were captured with a 40x objective lens at 5 minutes after FM1-43 treatment and whole cochleae was captured with 4x objective lens at 10 minutes, respectively. Images were obtained with an upright microscope (model TE300, Nikon, Tokyo, Japan) equipped with a digital camera (model DP73, Olympus, Tokyo, Japan). Gamma setting for green channel was adjusted equally for all images with using ImageJ software.

## Results

### Confirmation of *Ildr1* gene knockout

We obtained *Ildr1* knockout mice (*Ildr1* ^*tm1(KOMP)Wtsi*^) established by Knockout Mouse Project (Project ID:CSD31366) from Wellcome Trust Sanger Institute. The targeting strategy removed exons 3–5 of the *Ildr1* gene, which encodes angulin-2/ILDR1, and replaced these three exons with a *lacZ* cassette and a splice acceptor site (Fig. 1A) [20]. If splicing between exon 2 and exon 6 occurs in the targeted allele, a truncated angulin-2/ILDR1 protein with the mostly intact cytoplasmic domain, which contains the epitope for our anti-angulin-2/ILDR1 antibody, is generated. However, the truncated protein cannot be incorporated into the plasma membrane and is considered to lack all normal function as a tTJ-associated membrane protein because it lacks part of the extracellular domain and the entire transmembrane domain of angulin-2/ILDR1 encoded by exons 3–5. Southern blotting and genotyping PCR analyses supported that gene targeting occurred as predicted (Fig. 1B,C). Furthermore, immunofluorescence staining of the cochlea of homozygous mice with anti-angulin-2/ILDR1 antibody showed no signal at TCs in the organ of Corti, whereas clear staining for angulin-2/ILDR1 at TCs was detected in wild-type mice (Fig. 1D). From these results, we concluded that no functional angulin-2/ILDR1 protein is produced in an *Ildr1* ^*tm1(KOMP)Wtsi*^ mouse, which is designated as *Ildr1* ^*k-/-*^ in this study.

### Profound hearing loss in *Ildr1* ^*k-/-*^ mice

**Fig 2:**
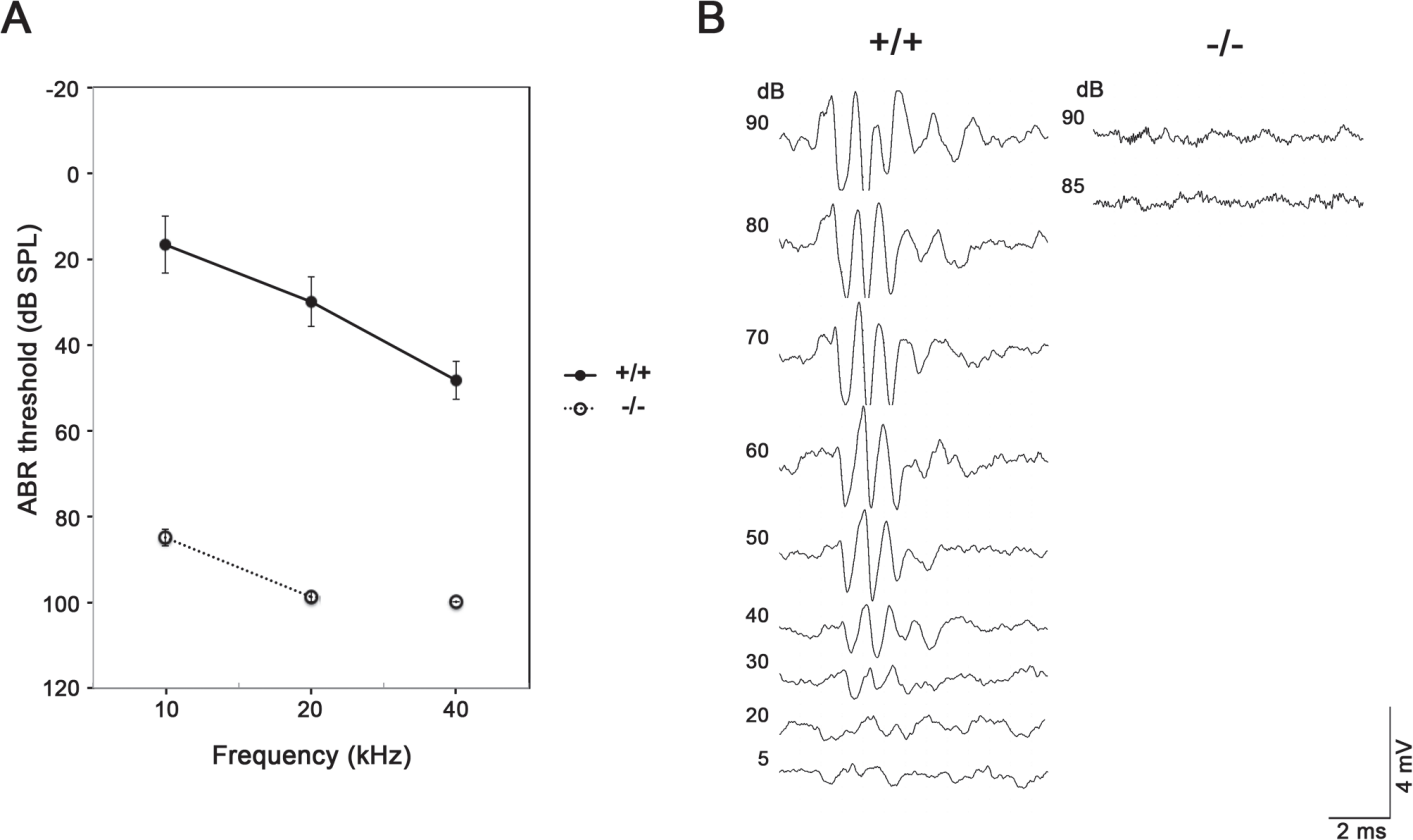
Hearing loss in *Ildr1* ^*k-/-*^ mice. A. Hearing thresholds at sound frequencies of 10, 20 and 40 kHz of wild-type (+/+) (n = 3) and homozygous (-/-) mice (n = 4) at P35. *Ildr1* ^*k-/-*^ mice showed increased thresholds (85- dB sound pressure level (SPL)) compared with wild-type mice (5–40 dB SPL). All average data on the graphs are shown as mean ± SEM. In all instances, P values were less than 0.01 and considered to be significant. B. ABRs to stimuli of 5–90 dB SPL at 20 kHz in P35 wild-type and *Ildr1* ^*k-/-*^ mice. Typical data for a wild-type mouse and an *Ildr1* ^*k-/-*^ mouse are shown. In wild-type mice, typical ABR waveform can be observed by small sound stimuli until 30dB. In contrast, *Ildr1* ^*k-/-*^ mice did not respond to large sound stimuli over 90dB.

To examine the cochlear function of *Ildr1* ^*k-/-*^ mice, auditory brain stem responses (ABRs) of postnatal day (P) 35 mice were measured using three different frequencies, 10 kHz, 20 kHz and 40 kHz. Compared with wild-type and *Ildr1* ^*k-/-*^ mice exhibited elevated ABR thresholds at all frequencies (Fig. 2A,B). The middle ear was aerated and no abnormalities wad found in tympanic membrane and ossicles in *Ildr1* ^*k-/-*^ mice at P35 (data not shown). These results show that *Ildr1* ^*k-/-*^ mice have profound sensorineural hearing loss.

### Progressive cochlear hair cells degeneration in *Ildr1* ^*k-/-*^ mice

**Fig 3:**
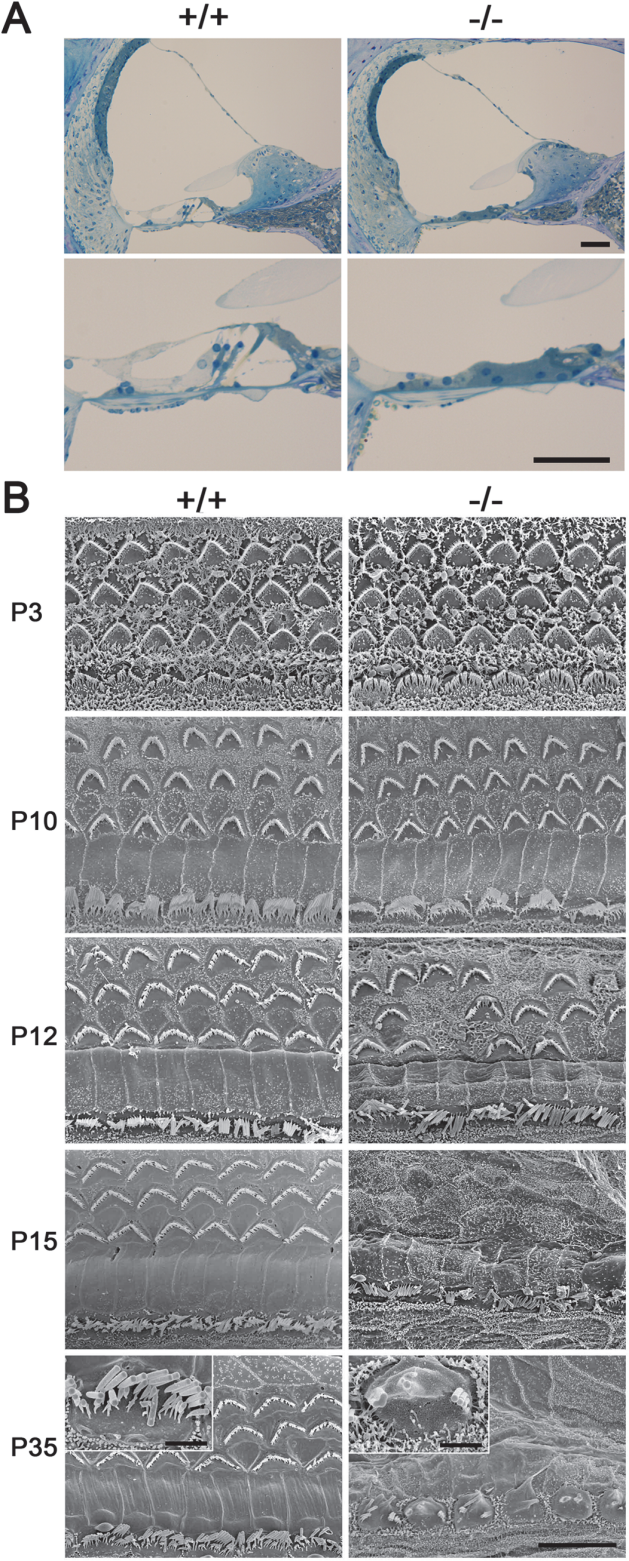
Morphological analyses of cochlea from *Ildr1* ^*k-/-*^ mice. A. Toluidine-blue-stained Epon semi-thin sections of the middle turn of cochlea in wild-type (+/+) and *Ildr1* ^*k-/-*^ (-/-) mice at P35. Higher magnification of organ of Corti is shown in the bottom panels. The structure of the organ of Corti in *Ildr1* ^*k-/-*^ mice was remarkably disrupted in contrast to wild-type. Bars, 50 μm. B. Scanning electron microscopic images of the surface of the organ of Corti in wild-type (+/+) and *Ildr1* ^*k-/-*^ (-/-) mice at various time points. The images were obtained from the middle turn. The surface structures were indistinguishable between wild-type and *Ildr1* ^*k-/-*^ mice at P3 and P10 while the OHCs of *Ildr1* ^*k-/-*^ mice were partially (P12) or completely (P15 and P35) lost and the IHCs were also affected at P35. Bar, 10 μm. Insets in P35 show close-up views of IHCs. Bar, 2 μm.

To determine the cause of hearing loss in *Ildr1* ^*k-/-*^ mice, we investigated the morphology of the cochlea by light microscopic observations of toluidine blue-stained thin sections. Although gross organization of the cochlea in *Ildr1* ^*k-/-*^ mice at P35 appeared normal compared with wild-type mice, we found that the organ of Corti in *Ildr1* ^*k-/-*^ mice remarkably collapsed, suggesting the loss of hair cells (Fig. 3A). Thus, we examined detailed morphology of hair cells in the cochlea by scanning electron microscopy and found that outer hair cells (OHCs) as well as inner hair cells (IHCs) were degenerated in *Ildr1* ^*k-/-*^ mice by P35: OHCs appeared to be lost completely and most of stereocilia were disorganized in IHCs (Fig. 3B). Next, we investigated the process of cochlear hair cell degeneration in the cochlea in *Ildr1* ^*k-/-*^ mice at various developmental stages. At P3 and P10, three rows of OHCs and a single row of IHCs appeared normal with well-developed stereocilia and no morphological difference of hair cells was detected compared with wild-type mice. At P12, part of OHCs were degenerated or lost, while IHCs still looked normal. By P15, almost all of OHCs were lost, while IHCs with stereocilia were still retained. (Fig. 3B). These observations indicate that hair cells develop normally in *Ildr1* ^*k-/-*^ mice but they gradually degenerate and are lost. These data suggest that postnatal degeneration of hair cells is the cause of hearing loss in *Ildr1* ^*k-/-*^ mice.

**Fig 4:**
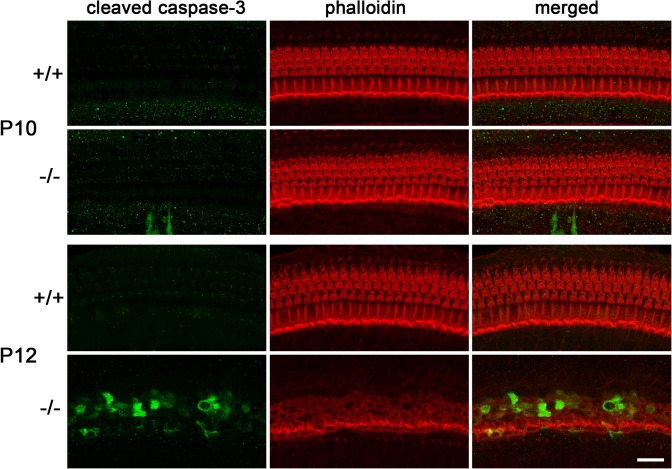
Apoptotic degenerative death of hair cells in *Ildr1* ^*k-/-*^ mice. Immunofluorescence microscopic images of the organ of Corti in the middle turn in wild-type (+/+) and *Ildr1* ^*k-/-*^ (-/-) mice at P10 and P12 using anti-cleaved caspase-3 antibody (green) and phalloidin (red). At P10, cleaved caspase-3 signal, which indicates apoptotic cells, was detected in the organ of Corti neither in wild-type nor in *Ildr1* ^*k-/-*^ mice. At P12, cleaved caspase-3 signal was hardly detected in wild-type tissue whereas prominent signals were detected in the OHCs and IHCs in *Ildr1* ^*k-/-*^ mice. Bar, 20 μm.

Next, we attempted to clarify the mechanism underlying degenerative loss of hair cells in postnatal *Ildr1* ^*k-/-*^ mice. To examine the involvement of apoptosis, isolated cochleae from wild-type and *Ildr1* ^*k-/-*^ mice at P10, when OHC appeared normal, and those at P12, when part of OHCs has already degenerated (Fig. 3B), were immunostained with an antibody against cleaved caspase-3, an indicator of apoptosis. Specimens were also labeled with fluorescent phalloidin, which stains filamentous actin, to identify hair cells. At P10, the organ of Corti of both wild-type and *Ildr1* ^*k-/-*^ mice contained no signal for cleaved caspate-3. At P12, a positive signal for cleaved caspase-3 was hardly detected throughout the organ of Corti in wild-type mice (Fig. 4). By contrast, cleaved caspase-3 signals were observed not only in OHCs but also IHCs in *Ildr1* ^*k-/-*^ mice (Fig. 4). These results indicate that the degenerative loss of hair cells in *Ildr1* ^*k-/-*^ mice is caused by apoptosis, which begins between P10 and P12, although we cannot deny the possible involvement of necrosis in this process.

**Fig 5:**
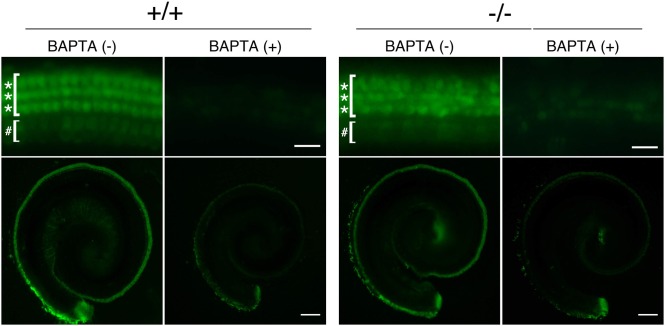
Mechanotransduction activity of neonatal *Ildr1* ^*k-/-*^ mice. Fluorescence microscopic images of the P3 mouse cochlea (*Ildr1* ^*k-/-*^, n = 3; wild-type, n = 5) exposed to 5 μM FM1-43 for 10 sec. Both wild-type (+/+) and *Ildr1* ^*k-/-*^ (-/-) mouse IHCs (#) and OHCs (*) showed robust uptake of FM1-43 with green signals, whereas both wild-type and *Ildr1* ^*k-/-*^ mouse hair cells pretreated with 5 mM BAPTA did not take up FM1-43. No remarkable differences were observed between wild-type and *Ildr1* ^*k-/-*^ mice. The lower panels contain lower magnification images of the upper panels. Bars: 200 μm, upper panels; 20 μm, lower panels.

To evaluate the function of morphologically normal neonatal hair cells of *Ildr1* ^*k-/-*^ mice before degeneration (P3), we examined FM1-43 uptake (Fig. 5). The process of mechanotransduction activity, the main function of sensory hair cells, is initiated by the opening of cation channels near the tips of stereocilia [21], which can be visualized by an uptake of a styryl fluorescent dye, FM1-43 [22,23]. As shown in Fig. 5, we observed that FM1-43 uptake into OHCs and IHCs was not affected throughout the cochlea in *Ildr1* ^*k-/-*^ mice, indicating that the hair cells of the *Ildr1* ^*k-/-*^ mice develop normally and have functional mechanotransduction channels at least until P3. This result further suggests that degeneration rather than maldevelopment of hair cells is the primary cause of hearing impairments in the *Ildr1* ^*k-/-*^ mice.

### Compensatory accumulation of angulin-1/LSR at TCs of the organ of Corti in *Ildr1* ^*k-/-*^ mice

**Fig 6:**
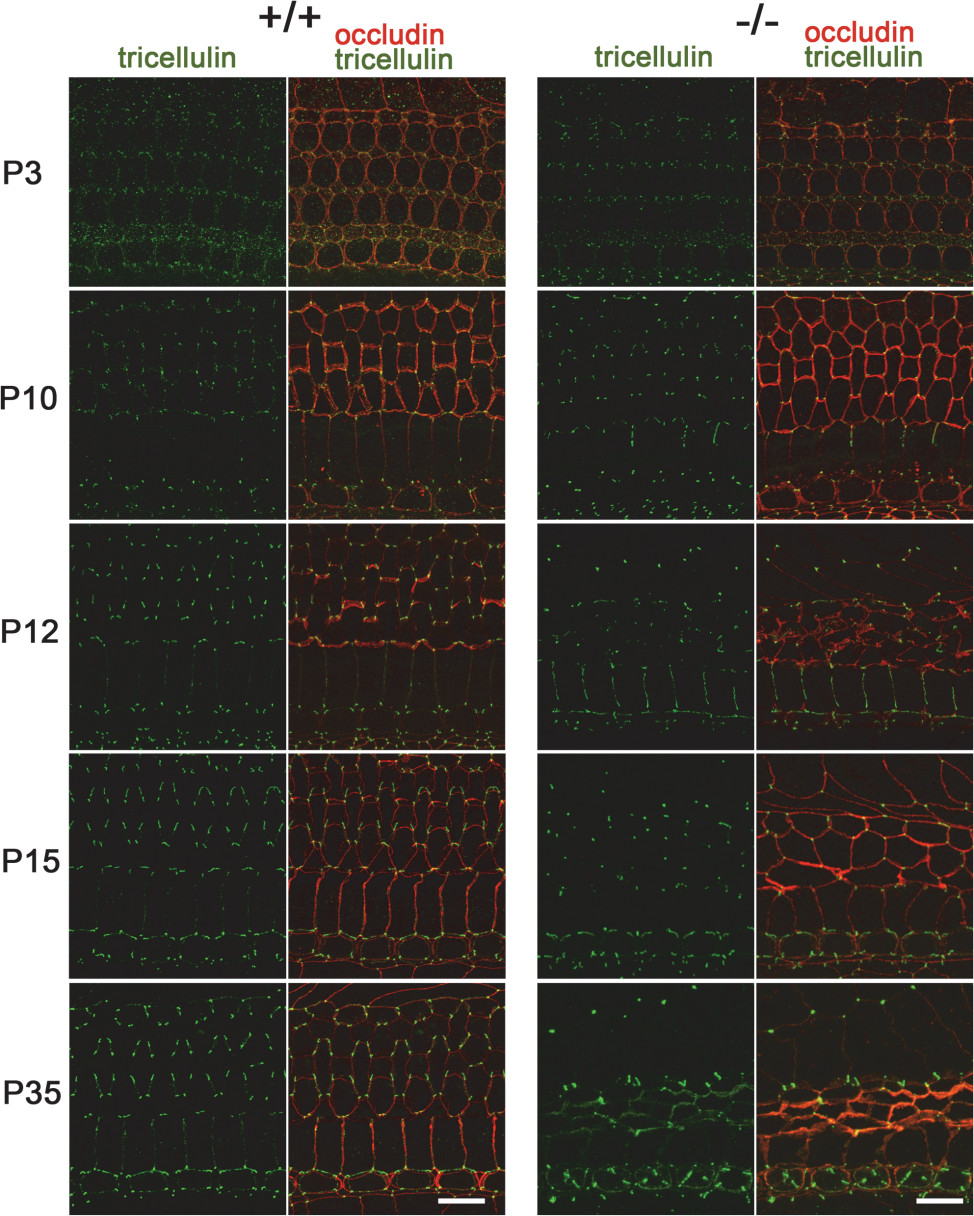
Localization of tricellulin in the organ of Corti in *Ildr1* ^*k-/-*^ mice. Immunofluorescence microscopic images of the organ of Corti in the middle turn in wild-type (+/+) and *Ildr1* ^*k-/-*^ (-/-) mice at various time points using anti-tricellulin mAb (green) and anti-occludin pAb (red). Tricellulin was concentrated at TCs in the organ of Corti in both wild-type and *Ildr1* ^*k-/-*^ mice at every time point, although its staining patterns from P12-35 in *Ildr1* ^*k-/-*^ mice are different from those in wild-type mice probably because of the disorganization of the organ of Corti by the hair cell loss. At least two wild-type mice and *Ildr1* ^*k-/-*^ mice were analyzed for each time point and the consistent results were obtained. Bars, 10 μm.

**Fig 7:**
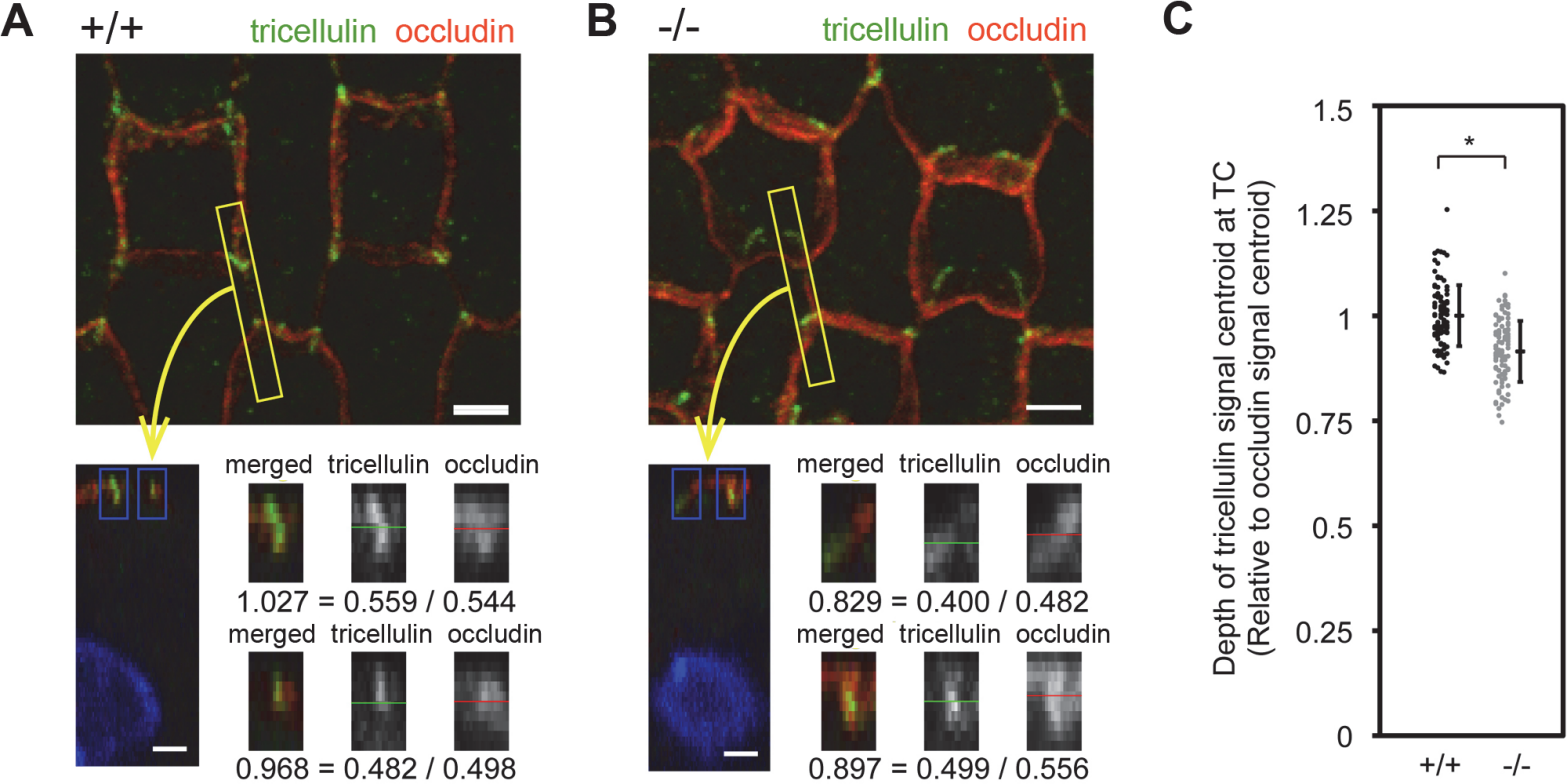
Change in the distribution of tricellulin along the length of TCs in the organ of Corti in *Ildr1* ^*k-/-*^ mice. A–B, Immunofluorescence microscopic images of the organ of Corti in the middle turn in wild-type (A) and *Ildr1* ^*k/-*^ (B) mice at P10 using anti-tricellulin (green) and anti-occludin (red) antibodies. A z-view of the area indicated by the yellow rectangle is also shown (lower left panels). Centroids of the fluorescence signals of tricellulin and occludin at TCs (blue rectangles) were determined using ImageJ software and the relative depths of the tricellulin signals were calculated by dividing by the depths of the occludin signals. Bars, 2 μm. C. Depths of tricellulin signals relative to occludin signals at 100 TCs were determined from two mice. Error bars indicate s.d. *p<0.005 (Student's t-test). Note that tricellulin was distributed more basolaterally within TCs in *Ildr1* ^*k/-*^ mice (-/-) compared with wild-type mice (+/+).

**Fig 8:**
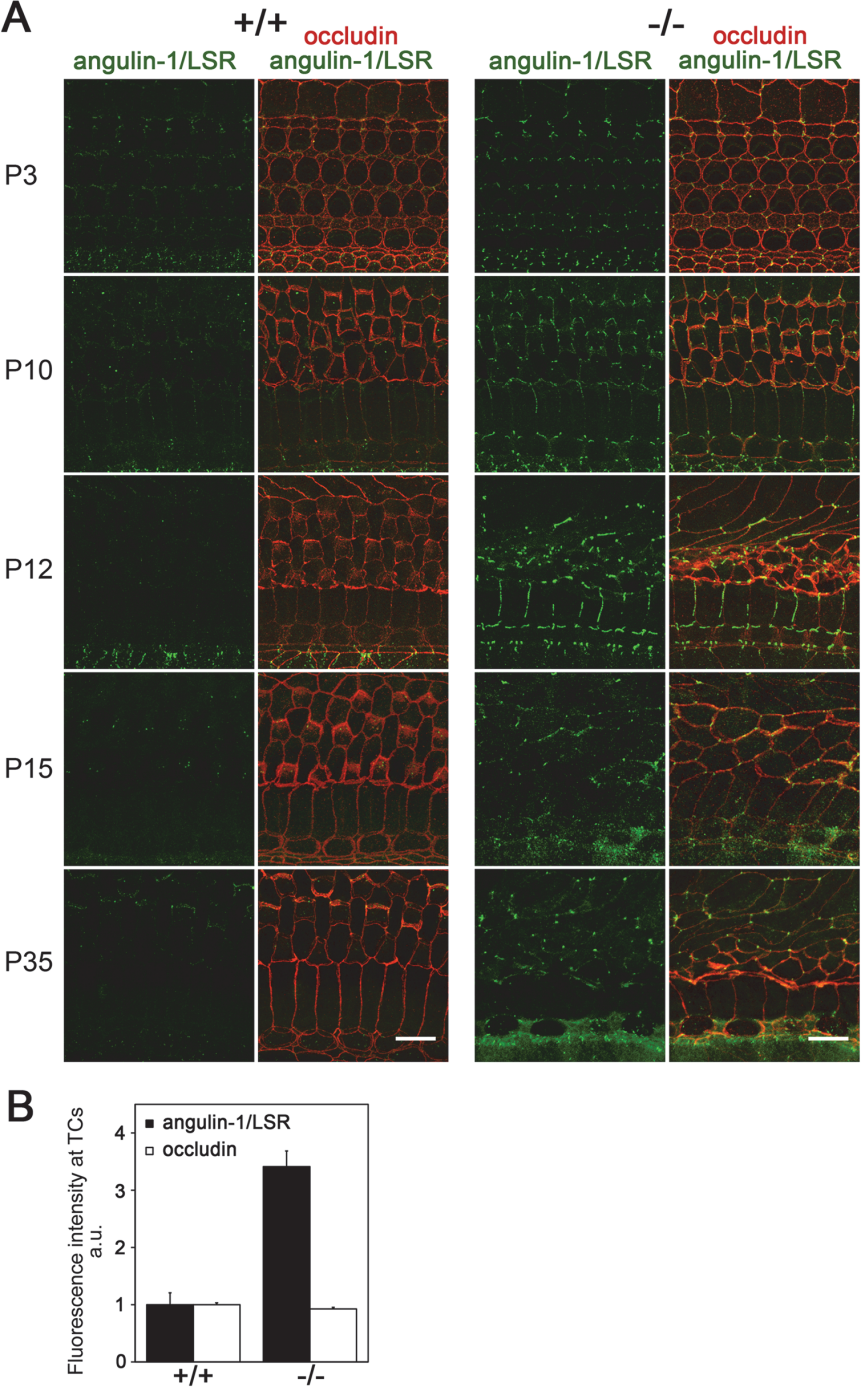
Compensatory accumulation of angulin-1/LSR in the organ of Corti in *Ildr1* ^*k-/-*^ mice. A. Immunofluorescence microscopic images of the organ of Corti in the middle turn in wild-type (+/+) and *Ildr1* ^*k-/-*^ (-/-) mice at various time points using anti-angulin-1/LSR mAb (green) and anti-occludin pAb (red). Note that the angulin-1/LSR signals are evident at TCs in the organ of Corti in *Ildr1* ^*k-/-*^ mice, but not in wild-type mice. B. The fluorescence intensities of angulin-1/LSR and occludin at TCs in the organ of Corti in wild-type mice and *Ildr1* ^*k/-*^ mice at P10 were quantified. Error bars indicate s.d. *p<0.005 (Student’s t-test). Note that the intensity of angulin-1/LSR was significantly up-regulated at TCs in *Ildr1* ^*k/-*^ mice, whereas that of occludin was unchanged. At least two wild-type mice and *Ildr1* ^*k-/-*^ mice were analyzed for each time point and consistent results were obtained. Bars, 10 μm.

Using immunofluorescence staining, we showed that angulin-2/ILDR1 is the dominant angulin at TCs in the mouse organ of Corti [9]. We also reported in a cultured epithelial model that all of the DFNB49-associated tricellulin mutant proteins could not be recruited to TCs by angulin-2/ILDR1, while most of DFNB42-associated angulin-2/ILDR1 mutant proteins showed impaired localization at TCs [9]. Furthermore, *Tric* ^*R497X/R497X*^ mice, which mimicked one of the *TRIC* mutations observed in DFNB49, exhibited profound deafness with postnatal hair cell degeneration and the C-terminus-truncated tricellulin protein encoded by this mutation could not be detected at TCs in these mice [15]. Taken together, these previous observations suggest the hypothesis that the mislocalization of tricellulin by the lack of angulin-2/ILDR1 around hair cells is the cause of deafness with hair cell degeneration in *Ildr1* ^*k-/-*^ mice. To evaluate this hypothesis, we analyzed the localization of tricellulin in the organ of Corti in various postnatal developmental stages of *Ildr1* ^*k-/-*^ mice by immunofluorescence staining. To our surprise, tricellulin was highly concentrated at TCs in the organ of Corti from P3 to P35 in *Ildr1* ^*k-/-*^ mice, although its staining patterns from P12-35 are different from those in wild-type mice probably because of the disorganization of the organ of Corti by the hair cell loss (Fig. 6). A detailed analysis of tricellulin localization along the depth of TCs in the organ of Corti by comparing the immunofluorescence signals of tricellulin with those of occludin, which delineates the entire length of TCs, showed that tricellulin was distributed to the more basal side of TCs in *Ildr1* ^*k-/-*^ mice than in wild-type mice at P10 (Fig. 7). This tricellulin localization at TCs in *Ildr1* ^*k-/-*^ mice implied that there might be compensation for the loss of angulin-2/ILDR1 by another member of the angulin family. Immunofluorescence staining revealed that angulin-1/LSR was clearly detected at TCs in the organ of Corti in *Ildr1* ^*k-/-*^ mice, whereas angulin-1/LSR was mostly undetectable at TCs in wild-type mice (Fig. 8A). Quantitative analyses showed that the mean fluorescence intensity of angulin-1/LSR at TCs in the organ of Corti was more than three-fold stronger in *Ildr1* ^*k-/-*^ mice than in wild-type mice at P10, whereas no significant change in the mean fluorescence intensity of occludin at the same TCs was observed between the two groups (Fig. 8B). These results suggest that the compensatory accumulation of angulin-1/LSR mediates localization of tricellulin at TCs in *Ildr1* ^*k*-/-^ mice, and that angulin-2/ILDR1 has a specific function that cannot be substituted by angulin-1/LSR in hearing.

## Discussion

Mutations of *ILDR1* cause human autosomal recessive deafness DFNB42. In the present study, we have demonstrated that targeted disruption of mouse *Ildr1* results in profound hearing loss accompanied by postnatal hair cell degeneration in mice in spite of a compensated accumulation of angulin-1/LSR at TCs. The hair cells of *Ildr1* deficient mice once developed normally in their morphology and function, but degenerated at least partly via apoptosis. This suggests that the loss of cochlear hair cells is the likely main cause of deafness DFNB42 in human patients.

The cochlea is responsible for hearing in the inner ear and consists of two types of fluid compartments filled with perilymph and endolymph [18]. Perilymph consists of low concentration of K^+^ and high concentration of Na^+^ similar to general extracellular fluid, whereas endolymph has a unique electrolyte concentration with high K^+^ and low Na^+^. In addition, the endolymph has a high positive electric potential called the endocochlear potential (EP). The high concentration of K^+^ and high EP of endolymph drive the influx of K^+^ ion into hair cells through ion channels upon mechanical stimulus of sound, which is then transmitted to the auditory neuron [18].

Compartmentalization of endolymph and perilymph to maintain their electrolyte contents and the EP requires strong epithelial barrier function, in which TJs and tTJs must play crucial roles in occlusion of the paracellular pathway. TJs of the organ of Corti in mice consist of various types of claudins, including claudin-14 and claudin-9 [24]. Mutations in *CLDN14*, which encodes claudin-14, cause non-syndromic hereditary deafness DFNB29 (MIM 614035) in human [25] and targeted disruption of *Cldn14* in mice results in hearing loss with rapid degeneration of OHCs and slower degeneration of IHCs [26]. Claudin-9 mutant mice have hearing loss with hair cell degeneration and elevated K^+^ concentration in the perilymph [27]. In addition to these claudin-deficient mice, the *Tric* ^*R497X/R497X*^ mouse, a knock-in model which mimics one of the mutations identified in the DFNB49 pedigrees, showed profound hearing loss accompanied by postnatal hair cell loss: OHCs were partly lost at P16 and no OHCs and degenerated IHCs were retained at P35. In *Tric* ^*R497X/R497X*^ mice, the tTJ structure of hair cells and supporting cells was severely disrupted although the EP was normal [15]. During our preparation of this manuscript, Morozko et al. have reported profound deafness accompanied by hair cell loss without a change of EP in angulin-2/ILDR1-deficient mice [28]. Considering the intimate relationship between angulins and tricellulin which are protein partners [9], we assume that hair cell degeneration in *Tric* ^*R497X/R497X*^ mice and *Ildr1* ^*k-/-*^ mice is mediated by a similar mechanism, which might be slight impairment of the epithelial barrier function limited at the organ of Corti.

On the other hand, a detailed comparison between *Ildr1* ^*k-/-*^ mice and *Tric* ^*R497X/R497X*^ mice may provide new insights into the molecular mechanism underlying the establishment of the tTJ barrier. In *Tric* ^*R497X/R497X*^ mice, truncated tricellulin p.R497X protein was not detected at TCs, while angulin-2/ILDR1 was localized at TCs in the organ of Corti [15]. In *Ildr1* ^*k-/-*^ mice, however, tricellulin was clearly concentrated at TCs probably by the observed compensated accumulation of angulin-1/LSR at TCs, which has an ability to recruit tricellulin. If the only role of angulin-2/ILDR1 in the cochlea is the recruitment of tricellulin to TCs, deafness with hair cell degeneration in *Ildr1* ^*k-/-*^ mice may be caused by impaired tTJ function because of the loss of tricellulin, which was shown to impair the barrier function of epithelial cellular sheets *in vitro* [7]. However, this explanation seems unlikely because the apparent localization of tricellulin at TCs in *Ildr1* ^*k-/-*^ mice at P10, just before the onset of the apoptosis of hair cells. In addition to tricellulin recruitment, angulin-2/ILDR1 may contribute to the establishment of the epithelial barrier in the organ of Corti by defining the distribution of tricellulin within TCs, which was suggested by our data and also by Morozko et al [28]. It is also possible that this may occur through an unknown process that is not related to tricellulin. The latter possibility is supported by the fact that the phenotype of hair cell degeneration in *Ildr1* ^*k-/-*^ mice is significantly more severe than in *Tric* ^*R497X/R497X*^ mice: OHC degeneration was observed at P12 in *Ildr1* ^*k-/-*^ mice, whereas OHCs in *Tric* ^*R497X/R497X*^ mice appeared morphologically normal at P12 but showed degeneration by P16 [15].

In the EpH4 mouse mammary epithelial cells in which endogenous angulins were depleted by shRNA, exogenous expression of angulin-1/LSR and angulin-2/ILDR1 rescued the barrier function of the cellular sheet evaluated by transepithelial electrical resistance as well as paracellular flux of tracer molecules [9]. In this experiment, difference in the ability in epithelial barrier function between angulin-1/LSR and angulin-2/ILDR1 was not clear. However, in the same assay, angulin-3/ILDR2 did not sufficiently recover the barrier function of the cellular sheet, although angulin-3/ILDR2 rescued TC localization of tricellulin similar to angulin-1/LSR and angulin-2/ILDR1 [9]. These observations support the idea that each of the three angulin subtypes has a unique function in addition to a common role of tricellulin recruitment. The present study suggests a functional difference between angulin-2/ILDR1 and angulin-1/LSR in the formation and/or function of tTJs, which is an interesting issue to be pursued for the understanding of variations of tTJs.
